# Age of onset for increased dose-adjusted serum concentrations of antidepressants and association with sex and genotype: An observational study of 34,777 individuals

**DOI:** 10.1007/s00228-023-03611-3

**Published:** 2024-01-10

**Authors:** Kristine Tveit, Monica Hermann, Roy M. Nilsen, Susanna M. Wallerstedt, Arvid Rongve, Espen Molden, Kristine Hole

**Affiliations:** 1 Department of Health and Caring Sciences, Western Norway University of Applied Sciences, Bergen, Haugesund, Stord Norway; 2 Department of Pharmacology, Sahlgrenska Academy, University of Gothenburg, Gothenburg, Sweden; 3 HTA-Centrum, Sahlgrenska University Hospital, Gothenburg, Sweden; 4 Department of Research and Innovation, Helse Fonna, Haugesund Hospital, Haugesund, Norway; 5 Institute of Clinical Medicine, University of Bergen, Bergen, Norway; 6 Center for Psychopharmacology, Diakonhjemmet Hospital, Oslo, Norway; 7 Department of Pharmacy, University of Oslo, Oslo, Norway; 8 Department of Life Sciences and Health, Oslo Metropolitan University, Oslo, Norway

**Keywords:** Age, Antidepressant, Pharmacokinetic variability, Therapeutic drug monitoring

## Abstract

**Purpose:**

The aim of this study was to examine the age of onset for increased dose-adjusted serum concentrations (C/D ratio) of common antidepressant drugs and to explore the potential association with sex and *CYP2C19/CYP2D6* genotype.

**Methods:**

Serum concentrations and prescribed daily doses for citalopram, escitalopram, sertraline, venlafaxine and mirtazapine, and *CYP* genotypes, were obtained from a therapeutic drug monitoring (TDM) service. Segmented linear regression analysis was used to examine the relationship between age and antidepressant log C/D ratio in (i) all individuals, (ii) men and women, and (iii) CYP2D6/CYP2C19 normal metabolizers (NMs) and CYP2D6/CYP2C19 intermediate or poor metabolizers (IMs/PMs).

**Results:**

A total of 34,777 individuals were included in the study; *CYP* genotype was available for 21.3%. An increase in C/D ratio started at 44‒55 years of age. Thereafter, the increase progressed more rapidly for citalopram and escitalopram than for venlafaxine and mirtazapine. A doubled C/D ratio was estimated to occur at 79 (citalopram), 81 (escitalopram), 86 (venlafaxine), and 90 years (mirtazapine). For sertraline, only modest changes in C/D ratio were observed. For escitalopram and venlafaxine, the observed increase in C/D ratio started earlier in women than in men. The results regarding *CYP* genotype were inconclusive.

**Conclusion:**

The age-related increase in C/D ratio starts in middle-aged adults and progresses up to more than twofold higher C/D ratio in the oldest old. Sertraline seems to be less prone to age-related changes in C/D ratio than the other antidepressants.

## Introduction

Treatment with antidepressants is common in current healthcare, and older people are a major user group. When antidepressants are administered to people aged 65 years or older, they have on average 1.5–2 times higher serum concentrations than younger people using equal doses [1, 2]. Furthermore, serum concentrations above the recommended reference range have been reported to be twice as common in older people, despite being prescribed lower doses [3].

Therapeutic reference ranges of antidepressants are generally based on healthy adult populations, and may not be valid for older patients [4]. As older people, especially the frail, are particularly vulnerable to side effects of drugs, elevated serum concentrations may be problematic in this patient group [5]. We have recently reported that only modest dose reductions occur among patients ≥ 65 years over a 10-year period and that more than one third of these patients have serum concentrations above the recommended reference range [6]. However, using an age cut-off of 65 can be questioned, as it reflects the traditional age of retirement rather than age-related physiological changes, such as altered fat/muscle ratio and decreased kidney function [7]. Indeed, it may be reasonable to assume that increasing concentrations with the same dose can be a continuous process with increasing age. Yet, as far as we are aware, it is not known at which age a change in antidepressant concentration-to-dose (C/D) ratio appears, and how the C/D ratio develops over time with increasing age.

Furthermore, one could speculate that sex and genetic prerequisites may have implications for the concentrations achieved. Indeed, an association between sex and antidepressant pharmacokinetics has been described [8], and progressive age–related changes in C/D ratio could therefore be suspected to differ between men and women. Regarding pharmacogenetics, polymorphic cytochrome P450 (CYP) enzymes are important for the metabolism of antidepressants. For instance, CYP2C19 is involved in the metabolism of citalopram, escitalopram, and sertraline; CYP2D6 is one of several CYP enzymes involved in the metabolism of mirtazapine; and for venlafaxine, both CYP2D6 and CYP2C19 are important for the serum concentration of the drug and the active moiety [9–12]. The Clinical Pharmacogenetics Implementation Consortium (CPIC) guideline provides dose recommendations according to *CYP2D6* and *CYP2C19* genotype for all the abovementioned drugs except mirtazapine [9].

In this study, we aimed to examine the age of onset for increased dose-adjusted serum concentration (C/D) of antidepressant drugs and to explore the potential association with sex and *CYP2C19/CYP2D6* genotype.

## Methods

### Patients and samples

Data were obtained from a therapeutic drug monitoring (TDM) database at Diakonhjemmet Hospital, Oslo, Norway. This database contains information on serum concentration measurements from patients that are clinically followed up in primary or secondary health care. Serum concentration measurements from the period 2010–2020 were extracted for individuals, 18 years or older, referred to TDM for one of the following antidepressants: citalopram, escitalopram, sertraline, venlafaxine, and mirtazapine. Patient age, sex, prescribed antidepressant dose, and sampling time were also extracted from the TDM database. For the subgroup of patients who had performed *CYP2D6* and *CYP2C19* pharmacogenetic analyses, these results were also obtained. Finally, when TDM of interacting drugs was requested concurrently with the included antidepressants, this information was extracted. The interacting drugs included the CYP inducers phenobarbital, phenytoin, and carbamazepine, and the CYP inhibitors bupropion, fluvoxamine, fluoxetine, levomepromazine, and paroxetine. Information regarding comedication with other drugs than the abovementioned was not available.

Blood samples were excluded if not collected within 10–26 h after last drug intake, and if the serum concentration was outside the limits of quantification. Samples where no metabolite was detected were also excluded as this probably reflects poor compliance. Furthermore, to avoid TDM results affected by drug interactions, samples were excluded if the abovementioned CYP inducers/inhibitors were analyzed and detected in the sample. Also, for some patients, there were multiple samples, and to maximize data at older ages, all but the most recent sample were excluded.

#### Drug analyses

Serum concentrations of the antidepressants, including the main metabolites, were determined by validated and certified analytical methods developed for routine TDM analyses at the Center for Psychopharmacology, Diakonhjemmet Hospital, Norway. During the inclusion period, the analytical assays were modified due to renewal of the analytical instruments, and all modifications were cross-validated. Briefly, in the current ultra-performance liquid chromatography high-resolution MS (UPLC-HRMS) method, protein precipitation was performed with an acetonitrile-methanol mix. Purified serum samples were injected into a Vanquish UPLC system (Thermo Fisher Scientific, Waltham, MA, USA). Chromatographic separation of all compounds was achieved with an XBridge BEH C18 column (2.6 µm, 2.1 × 75 mm; Waters Corporation, Milford, MA, USA) by gradient elution with acetonitrile and ammonium acetate (pH = 4.8).

Using a QExactive Hybrid Quadropole-Orbitrap MS (Thermo Fisher Scientific), full scan data was acquired at a resolution of 70,000 within the 100–1500-Da scan range. The compounds were quantified in a full-scan acquisition mode, and *m/z* values were 325.17107 for citalopram and escitalopram, 311.15542 for desmethylcitalopram and desmethylescitalopram, 306.08108 for sertraline, 292.0654 for desmethylsertraline, 278.21146 for venlafaxine, 264.19581 for O-desmethylvenlafaxine, 266.16517 for mirtazapine, and 252.14952 for desmethylmirtazapine. The limits of quantification were 8–1000 nmol/L for citalopram/escitalopram, 5–400 nmol/L for desmethylcitalopram/escitalopram, 10–600 nmol/L for sertraline, 20–1200 nmol/L for desmethylsertraline, 10–800 nmol/L for mirtazapine, 10–500 nmol/L for desmethylmirtazapine, 50–4000 nmol/L for venlafaxine, and 75–4000 nmol/L for O-desmethylvenlafaxine. Intra- and inter-day validation parameters of imprecision and inaccuracy showed less than 15% deviation for all compounds. For venlafaxine, the serum concentration is presented as the active moiety, i.e., the sum of parent drug and active metabolite.

### Genotyping

Genotyping of *CYP2D6* included identification of the no function variant alleles CYP2D6*3 (rs35742686), CYP2D6*4 (rs3892097), CYP2D6*5 (gene deletion), and CYP2D6*6 (rs5030655); the decreased function alleles CYP2D6*9 (rs5030656), CYP2D6*10 (rs1065852), and CYP2D6*41 (rs28371725); and allele multiplication. Genotyping of *CYP2C19* included the no function alleles CYP2C19*2 (rs4244285), CYP2C19*3 (rs4986893), and CYP2C19*4 (rs28399504), and the increased function allele CYP2C19*17 (rs12248560). Analysis of variant alleles was performed using the TaqMan-based real-time PCR assays implemented for routine pharmacogenetic analyses at the Center for Psychopharmacology, Diakonhjemmet Hospital. To detect deletion or multiplication of *CYP2D6*, copy number analysis was performed with TaqMan Copy Number Assay targeting exon 9, using RNase P as endogenous control.

### Genotype-predicted phenotype

Patients who carried two no function alleles were defined as poor metabolizers (PMs). Patients carrying a no function allele combined with a decreased function allele, a normal function allele, or an increased function allele, and patients carrying two decreased function alleles, were defined as intermediate metabolizers (IMs). Those who presented two increased function alleles (CYP2C19*17/*17) or more than two CYP2D6*1 alleles were defined as ultrarapid metabolizers (UMs). For *CYP2D6,* individuals with gene duplication and a no function or decreased function allele were classified as having unknown *CYP2D6* genotype, due to lacking information regarding which allele was duplicated. The remaining individuals were classified as normal metabolizers (NMs). Individuals with the CYP2C19*1/*17 genotype were merged into the NM phenotype group in concordance with guidelines from the Dutch Pharmacogenetics Working Group [13].

### Statistics

The distribution of concentration-to-dose (C/D) ratios of the examined drugs were right skewed and therefore log transformed to better approach the normality assumption in linear regression analyses. For the same reason, C/D ratios are presented as geometric means.

Segmented linear regression analysis was used to examine the relationship between age and log C/D ratio for the studied antidepressants. This regression technique performs piecewise linear regressions, allowing estimation of the point where the regression line changes direction, i.e., the “breakpoint” (B) [14]. A starting value for age was chosen for the estimation of one potential breakpoint and two piecewise relations for each antidepressant. The starting values were obtained using Davies test evaluated at *k* = 100 points [14]. Sex and blood sampling time were included as covariates in the segmented regression analysis. The regression coefficients for the slope before and after the breakpoint B were denoted R1 and R2, respectively. Furthermore, we used 95% confidence interval as a measure of uncertainty for all estimates of B, R1, and R2.

The segmented regression analyses were performed for (1) all individuals, (2) men and women separately, and (3) CYP2D6/CYP2C19 NMs and CYP2D6/CYP2C19 IM/PMs separately. That is, for citalopram, escitalopram, and sertraline, segmented regression analyses were performed for CYP2C19 NMs and CYP2C19 IMs/PMs separately, while for mirtazapine, segmented regression analyses were performed for CYP2D6 NMs and CYP2D6 IMs/PMs separately. Since metabolism of venlafaxine depend on both CYP2D6 and CYP2C19, individuals with normal vs reduced CYP-metabolism were separated into combined CYP2D6 and CYP2C19 NMs and individuals with all combinations of CYP2D6 and CYP2C19 NM/IM/PM, except for CYP2D6 and CYP2C19 NMs. UMs of the respective CYP enzyme(s) for each antidepressant were excluded, because the UM subgroups were too small to be included in the segmented regression analyses.

Median with interquartile range (IQR) was used to describe central tendency of age, dose, and sampling time because of extreme values in the study sample.

Stata IC version 16 (StataCorp llc, Statistical Software, College Station, TX, USA) was used for descriptive analyses, while R was used for estimation of breakpoint (B) and regression coefficients (R1 and R2) by segmented regression analyses, including Davies test.

### Ethical considerations

The study was approved by the Regional Committee for Medical and Health Research Ethics (ref. 2018/655) and the Hospital Investigational Review Board.

## Results

### Patients and samples

**Fig. 1:**
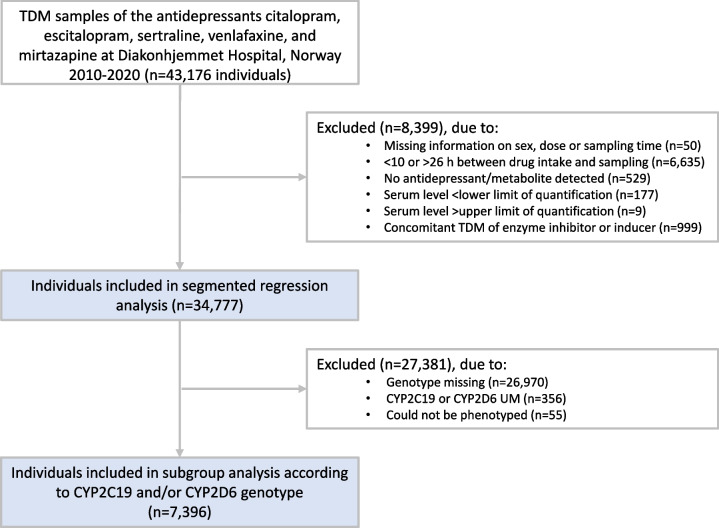
Flowchart illustrating patient inclusion and exclusion

In all, 34,777 unique individuals were included in the analyses, where 12,385 (36%) were men. In the subgroup analysis according to genotype, 7,396 unique individuals were included, whereof 2740 (37%) were men. A flowchart of the inclusion and exclusion process is presented in Fig. 1, and patient characteristics for the different antidepressants are presented in Table 1.

**Table 1:** Patient characteristics according to antidepressant drug

**Antidepressant**	**Sex**	***n*** (%)	**Age, yrs, median (IQR), range**	**Dose, mg, median (IQR)**	**Sampling time, h, median (IQR)**	**Genotype**	
						Data available *n* (%)	NMs *n* (% of genotyped individuals)
Citalopram	Men	698 (31)	54 (41–71), 19–97	20 (20–30)	23 (16–24)	102 (15)	67 (66)
	Women	1577 (69)	59 (43–80), 18–105	20 (15–30)	23 (16–24)	242 (15)	162 (67)
Escitalopram	Men	5770 (35)	45 (33–60), 18–103	15 (10–20)	23 (16–24)	1182 (20)	831 (70)
	Women	10,882 (65)	46 (32–65), 18–104	10 (10–20)	23 (16–24)	1986 (18)	1318 (66)
Sertraline	Men	1470 (33)	42 (29–55), 18–93	100 (50–150)	22 (14–24)	349 (24)	250 (72)
	Women	2991 (67)	44 (30–60), 18–99	100 (50–100)	22 (14–24)	682 (23)	473 (69)
Venlafaxine	Men	2188 (37)	47 (35–59), 18–96	150 (150–225)	23 (14–24)	464 (21)	200 (24)
	Women	3681 (63)	49 (36–64), 18–99	150 (112.5–225)	23 (15–24)	824 (22)	307 (37)
Mirtazapine	Men	2259 (41)	56 (40–73), 18–103	30 (30–45)	13 (12–14)	643 (28)	353 (55)
	Women	3261 (59)	66 (46–82), 18–106	30 (15–45)	13 (12–14)	1565 (48)	827 (53)

*IQR* interquartile range, *NMs* normal metabolizers (regarding CYP2C19 for citalopram, escitalopram, and sertraline; CYP2C19 and CYP2D6 for venlafaxine; and CYP2D6 for mirtazapine)

### Changes in C/D ratio related to age

**Fig. 2:**
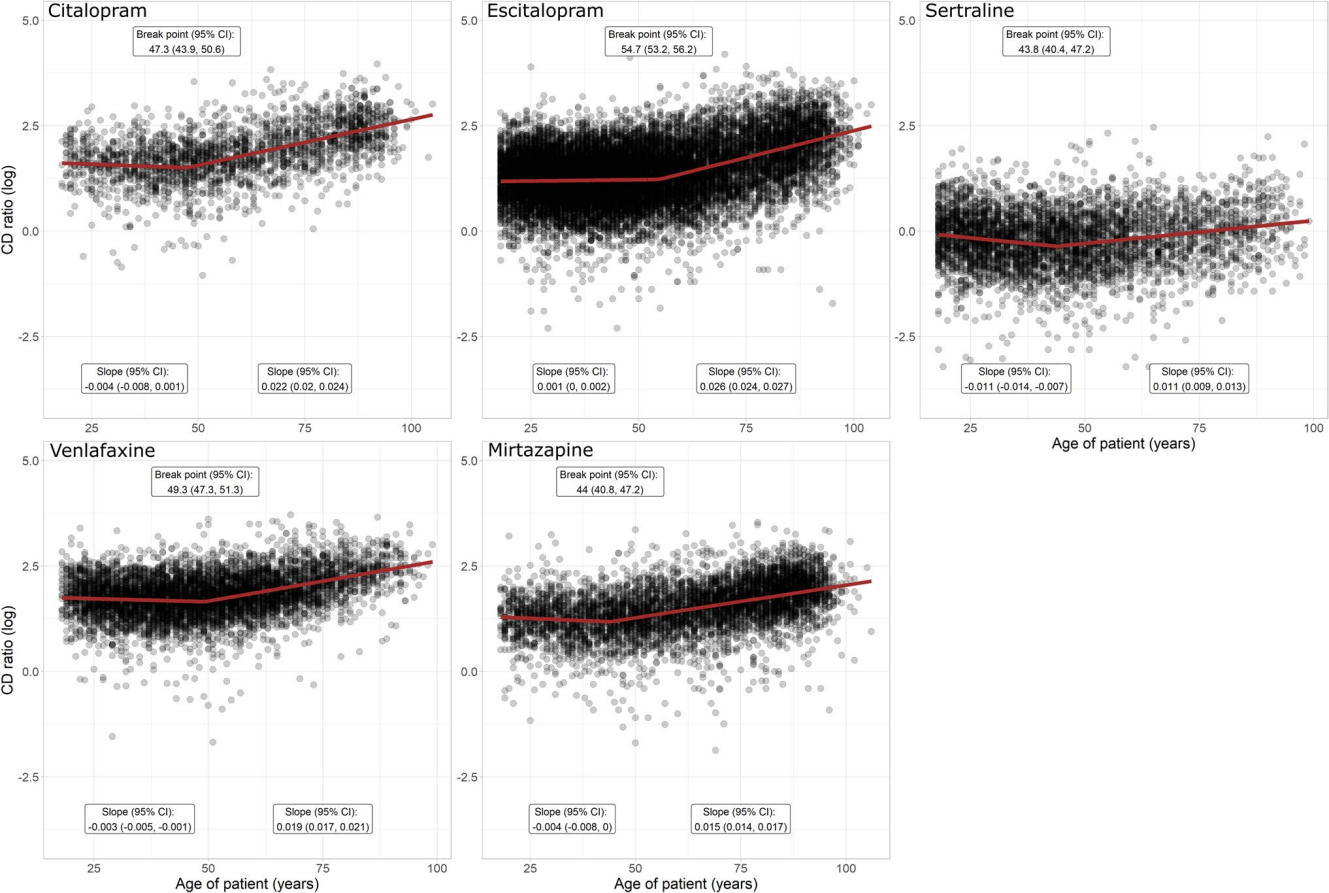
Segmented regression analysis of patient age versus concentration-to-dose (CD)-ratio ((nmol/L)/mg) for citalopram (*n* = 2275), escitalopram (*n* = 16652), sertraline (*n* = 4461), venlafaxine (*n* = 5869), and mirtazapine (*n* = 5520). The break point identifies the age (years) where the C/D ratio starts to change direction, while the regression slopes identify the average annual change in C/D ratio before and after the break point on a log scale. CI, confidence interval

For the five examined antidepressants, the change in the C/D ratio started between the age of 44 to 55 years (Table 2). The age of onset for an increased C/D ratio is denoted B (breakpoint in the segmented regression analysis) and is illustrated in Fig. 2. R1 and R2 show the annual change in log C/D ratio before and after age B, respectively. For all studied antidepressants, the log C/D ratio before age B did not change with increasing age, except for sertraline where a small decline in log C/D ratio with increasing age was observed, followed by an increase in slope after age B (Fig. 2). After B, the C/D ratio increased more rapidly for citalopram and escitalopram than for venlafaxine and mirtazapine, and a doubled C/D ratio was estimated at 79 and 80 years of age, respectively (Table 2; Appendix 1). For venlafaxine and mirtazapine, a doubled C/D ratio was estimated at 86 and 90 years of age. The yearly increase in C/D ratio was smaller for sertraline than for the other antidepressants.

**Table 2:** Estimated age when antidepressant dose-adjusted serum concentration (C/D-ratio) is increased by 1.5-fold, twofold, and threefold

**Antidepressant**	**Parameters estimated from segmented regression**				**Log C/D ratio at B (nmol/L)/mg**^**e**^	**Standard C/D ratio at B (nmol/L)/mg**^**f**^	**Age at which the C/D ratio is increased by:**^**g**^		
	*A*^a^	B (95% CI)^b^	R1 (95% CI)^c^	R2 (95% CI)^d^			1.5-fold	twofold	threefold
Citalopram	1.84600	47.3 (43.9, 50.6)	− 0.004 (− 0.008, 0.001)	0.022 (0.020, 0.024)	1.6568	5.2425	66	79	97
Escitalopram	1.49210	54.7 (53.2, 56.2)	0.001 (0.000, 0.002)	0.026 (0.024, 0.027)	1.5468	4.6964	70	81	97
Sertraline	0.45267	43.8 (40.4, 47.2)	− 0.011 (− 0.014, − 0.007)	0.011 (0.009, 0.013)	-0.0291	0.9712	81	107^h^	144^h^
Venlafaxine	2.20910	49.3 (47.3, 51.3)	− 0.003 (− 0.005, − 0.001)	0.019 (0.017, 0.021)	2.0612	7.8554	71	86	107^h^
Mirtazapine	1.59860	44.0 (40.8, 47.2)	− 0.004 (− 0.008, 0.000)	0.015 (0.014, 0.017)	1.4226	4.1479	71	90	117^h^

*CI* confidence interval

^a^Intercept A for the segmented regression

^b^Breakpoint B in the segmented regression analysis (identifies the age where the log C/D ratio starts to change direction in the segmented regression)

^c^Regression slope R1 for the first segmented line before breakpoint B (identifies the annual change in log C/D ratio before breakpoint B)

^d^Regression slope R2 for the second segmented line after breakpoint B (identifies the annual increase in log C/D ratio after breakpoint B)

^e^Estimated log C/D ratio at age B using the first segmented regression line (see Appendix 1)

^f^Estimated C/D ratio at age B on standard scale by exponentiating the log C/D ratio

^g^Estimated using the formula: ${\text{Age}}= \frac{{\text{log}} \left(1+\frac{pct}{100}\right)+{R}_{2}\cdot B}{{R}_{2}}$, where $pct$ indicates the percentage increase in standard C/D ratio from breakpoint B (see Appendix 1)

^h^The estimated age is outside the range of the included data

### Differences between men and women

For escitalopram and venlafaxine, the age-related change in C/D ratio was observed 5–6 years earlier for women when compared with men. However, the increase in C/D ratio after age B was smaller in women when compared with men for both escitalopram and venlafaxine. Similar findings were observed for citalopram, despite somewhat overlapping confidence intervals (Table 3). For sertraline, the increase in C/D ratio seems to start later for women compared with men, but this finding is uncertain due to wide confidence intervals (Table 3). No difference between men and women was observed for mirtazapine (Table 3).

**Table 3:** Estimated breakpoint (B) and regression slopes (R) for the association between age and dose-adjusted serum concentration (C/D ratio) according to antidepressant and sex

**Antidepressant**	**Subgroup**	**Total**	**Parameters estimated from segmented regression, mean (95% confidence interval)**		
			B^a^	R1^b^	R2^c^
Citalopram	Male	698	53 (47, 59)	0.001 (− 0.006; 0.007)	0.025 (0.021; 0.030)
	Female	1577	45 (41; 48)	− 0.007 (− 0.014; − 0.001)	0.021 (0.019; 0.023)
Escitalopram	Male	5770	56 (53; 59)^d^	0.005 (0.003; 0.007)^d^	0.027 (0.025; 0.030) ^d^
	Female	10,882	51 (49; 52)	− 0.003 (− 0.004; − 0.001)	0.024 (0.022; 0.025)
Sertraline	Male	1470	37 (31; 44)	− 0.010 (− 0.019; − 0.001)	0.009 (0.006; 0.012)
	Female	2991	44 (40; 48)	− 0.014 (− 0.018; − 0.009)	0.011 (0.009; 0.014)
Venlafaxine	Male	2188	53 (50; 56)^d^	− 0.001 (− 0.004; 0.001)	0.023 (0.019; 0.026) ^d^
	Female	3681	47 (45; 50)	− 0.004 (− 0.007; − 0.001)	0.018 (0.016; 0.019)
Mirtazapine	Male	2259	45 (40; 51)	− 0.002 (− 0.008; 0.003)	0.015 (0.013; 0.017)
	Female	3261	44 (39; 48)	− 0.006 (− 0.011; 0.000)	0.016 (0.015; 0.017)

^a^Breakpoint B in the segmented regression analysis (identifies the age where the log C/D-ratio starts to change direction in the segmented regression)

^b^Regression slope R1 for the first segmented line before breakpoint B (identifies the annual change in log C/D ratio before breakpoint B)

^c^Regression slope R2 for the second segmented line after breakpoint B (identifies the annual increase in log C/D ratio after breakpoint B)

^d^The 95% confidence intervals for men and women did not overlap

### Differences related to genotype

For individuals with genotype available, the results regarding age of onset for increased C/D ratio were inconclusive. However, in spite of overlapping confidence intervals, a trend towards 6 to 9 years earlier increase in C/D ratio was observed for CYP2C19 IMs/PMs on escitalopram and combined CYP2C19/CYP2D6 IMs/PMs on venlafaxine (Table 4).

**Table 4:** Estimated breakpoint (B) and regression slopes (R) for the association between age and dose-adjusted serum concentration (C/D ratio) according to antidepressant and pharmacogenetic profile

**Antidepressant**	**Genotype-predicted phenotype**	**Included patients (% female)**	**Segmented regression analyses, mean (95% confidence interval)**		
			B^a^	R1^b^	R2^c^
Citalopram	CYP2C19 NMs	229 (71)	48 (41; 55)	− 0.013 (− 0.027; 0.001)	0.026 (0.019; 0.033)
	CYP2C19 IMs/PMs	115 (70)	50 (27; 73)	0.003 (− 0.016; 0.021)	0.019 (0.008; 0.029)
Escitalopram	CYP2C19 NMs	2149 (61)	52 (47; 57)	0.000 (− 0.003; 0.004)	0.021 (0.017; 0.025)
	CYP2C19 IMs/PMs	1019 (66)	46 (39; 53)	− 0.003 (− 0.009; 0.003)	0.017 (0.013; 0.020)
Sertraline	CYP2C19 NMs	723 (65)	46 (39; 53)	− 0.016 (− 0.025; − 0.007)	0.013 (0.008; 0.019)
	CYP2C19 IMs/PMs	308 (68)	44 (36; 52)	− 0.020 (− 0.032; − 0.009)	0.011 (0.004; 0.018)
Venlafaxine	CYP2D6 and CYP2C19 NMs	507 (61)	57 (50; 64)	0.001 (− 0.004; 0.005)	0.023 (0.015; 0.030)
	CYP2D6/CYP2C19 IMs/PMs	781 (66)	48 (42; 55)	− 0.004 (− 0.001; 0.022)	0.017 (0.012; 0.021)
Mirtazapine	CYP2D6 NMs	827 (57)	38 (32; 44)	− 0.014 (− 0.027; -0.001)	0.013 (0.010; 0.016)
	CYP2D6 IMs/PMs	738 (61)	44 (37; 51)	− 0.010 (− 0.021; 0.000)	0.015 (0.012; 0.019)

*NMs* normal metabolizers, *IMs* intermediate metabolizers, *PMs* poor metabolizers

^a^Breakpoint B in the segmented regression analysis (identifies the age where the log C/D-ratio starts to change direction in the segmented regression)

^b^Regression slope R1 for the first segmented line before breakpoint B (identifies the annual change in log C/D ratio before breakpoint B)

^c^Regression slope R2 for the second segmented line after breakpoint B (identifies the annual increase in log C/D ratio after breakpoint B)

## Discussion

Examining almost 35,000 serum concentration measurements from unique individuals in a TDM service in Norway, we observed that the C/D ratio for the studied antidepressants started to increase between the age 44 to 55 years. For citalopram, escitalopram, venlafaxine, and mirtazapine, the C/D ratio was increased with 100% at the age of 79 to 90 years. For sertraline, only modest changes in C/D ratio were observed, indicating that age is less important for the pharmacokinetics of sertraline than the other examined antidepressants.

The steepest annual increase in C/D ratio was found for citalopram and escitalopram, which were estimated to have threefold higher serum concentration at the age of 97 compared with middle-aged adults receiving the same doses. Currently, among the antidepressants examined, citalopram and escitalopram are the only drugs where recommendations on dose reduction for older people are included in the summary of product characteristics (SPC). In 2011, a recommendation was added to reduce the daily dose of citalopram/escitalopram by 50% to people ≥ 65 years, after post-marketing reports of cardiotoxic effects and increased attention regarding dose-related risk of QT-prolongation [15, 16]. Our results show that increasing concentration with the same dose is a continuous process and that instead of a cut-off at 65 years, dose reductions could be considered continuously, in particular at the oldest ages.

At present, for venlafaxine and mirtazapine, there are no recommendations regarding dose reduction in older people. Studies that examined the correlation between antidepressant doses and QT-prolongation concluded that the effect for venlafaxine and mirtazapine was negligible [17, 18]. However, an association between QTc prolongation and high serum concentration has been demonstrated for older people using venlafaxine, and cardiotoxic effect is reported in CYP2D6 PMs and in patients using CYP2D6 inhibitors [19, 20]. A case report of mirtazapine-induced long QT syndrome has also been described in an older patient using low-dose (15 mg) mirtazapine, and both venlafaxine and mirtazapine are classified as drugs with possible increased risk of QT time [21, 22]. It has been reported that a prolonged QT interval of ≥ 500 ms is associated with considerably increased mortality in hospitalized patients independent of comorbidity [23]. Older people are at increased risk not only because of the higher age but also because of the higher occurrence of hypokalemia and use of antiarrhythmic drugs [24]. Additionally, old age and high serum concentrations are risk factors for side effects such as hyponatremia and serotonin syndrome, as well as side effects that are more difficult to quantify [25–27]. Side effects of antidepressants may be difficult to unveil because of the similarity with the disease for which they are prescribed for, i.e., increased restlessness, anxiety, and aggression. Based on our findings of an increasing C/D ratio with increasing age, combined with the general higher risk of side effects in older people, TDM may be a valuable tool to personalize treatment with venlafaxine and mirtazapine for older patients.

When examining differences between men and women, the age-related change in C/D ratio started 5 to 6 years earlier for women compared with men using venlafaxine or escitalopram. However, when the C/D ratio starts to increase, the annual age-related increase in C/D ratio for venlafaxine seems to be higher in men compared with women. This indicates that eventually, within the oldest old, perhaps because of age-related pharmacokinetic effects, differences in C/D ratio related to sex may even out [28]. Furthermore, this also means that age may have an independent effect on the C/D ratio of antidepressants and that older people are in general at increased risk of high serum concentration. Sex hormones play a role in the pharmacokinetic variability of psychotropic drugs [29]. Potential use of contraceptives, menopausal changes in estrogen, and possible estrogen substitution make the assessments of age-related changes in C/D ratios more complex in women than in men.

It is important to note that the present results are exclusively based on the observed changes in the systemic serum concentrations with increasing age. Both differences in the distribution of drugs into the central nervous system (CNS) and pharmacodynamic differences between older and younger individuals may result in altered effects, or risk of side effects, despite similar serum concentrations. For instance, increased permeability of the blood–brain barrier and decreased activity of the P-glycoprotein may contribute to a higher CNS drug disposition and risk of side effects despite stable serum levels [30–34]. Concerning pharmacodynamic changes at old age, a general reduction in homeostatic mechanisms, as well as changes in levels of neurotransmitters, serotonin receptors, and transporters, is associated with a higher risk of side effects [30, 35, 36]. Altogether, based on the existing knowledge of age-related changes in pharmacokinetics and pharmacodynamics and the increased risk of side effects in older people, dose reductions to older people seem reasonable.

There are some limitations of this study that should be considered. The observed increase in C/D ratio with increasing age may be associated with impaired renal function. For example, the clearance of O-desmethylvenlafaxine is reported to decrease in renally impaired patients [37]. However, we did not have information regarding renal function for the patients. For the same reason, frailty and potential reductions in hepatic clearance could not be considered [7, 38]. Furthermore, smoking may increase CYP1A2 metabolism of mirtazapine, but smoking status was not available for the included patients [39]. Also, despite exclusion of samples to avoid interference caused by drug interactions, we cannot guarantee that such bias is avoided as drugs for which TDM had not been requested could not be taken into account. Unfortunately, we did not have access to complete medication lists of the included patients. Increasing age is associated with increasing number of drugs, which could lead to more drug interactions. However, we were not able to separately describe the effect that reduced renal and hepatic function, comorbidities, or increased number of concomitant drugs may have had on the increase in C/D ratio. Indeed, the regression slopes for citalopram and escitalopram were particularly steep, and it cannot be excluded that the extensive use of the CYP2C19 inhibitor omeprazole at high ages may have contributed to this finding [40]. Furthermore, when considering differences between individuals with normal versus reduced metabolic capacity, the sample sizes were not large enough to perform separate analyses for individuals with IM and PM genotype-predicted phenotype. Nevertheless, a strength of the present study is the large number of individuals which made it possible to examine not only differences in C/D ratio between older and younger individuals but also the age where these changes begin and how these changes proceed with increasing age.

## Conclusion

The present study, based on TDM data, shows that the age-related increase in antidepressant C/D ratio on average appeared already in middle-aged adults and progressed up to more than doubled C/D ratios in the oldest old. Sertraline seems to be less prone to age-related changes in C/D ratio than the other antidepressants. Although the therapeutic reference range is large for these substances, the increasing C/D ratio by age could be worthwhile considering in the treatment of older patients.

## Data Availability

The data of this study are available from the corresponding author upon reasonable request.

## Appendix 1

Formula for calculation of increase in dose-adjusted serum concentration (C/D ratio).

1. Let $B$ represent the age where the C/D ratio starts to change direction on the log scale in the segmented regression (the breakpoint). At any age $x\le B$ (first segmented line), we have the following regression line describing the log C/D ratio:
  1 $$\log{(Y_1)}=A+R1\cdot x$$
  where $A$ is the intercept and $R1$ is the slope coefficient for age $x$.
2. We use Eq. 1 to estimate the log C/D ratio at the breakpoint age $B$:
  2 $${\text{log}}\left({Y}_{B}\right)= A + R1\cdot B$$
3. At any age $x \ge B$ (second segmented line), we have the following regression line describing the log C/D ratio:
  3 $$\log{(Y_x)} = A + R1\:\cdot B\: + R2 \cdot (x-B)$$
  where $R2$ is the slope coefficient for age $x$.
4. We then subtract Eq. 2 from 3, which provides us with the difference in log C/D ratio between age x and the breakpoint age B. The subtraction of equations further simplifies to:
  4 $${\text{log}}(\frac{{Y}_{x}}{{Y}_{B}})=R2\cdot (x-B)$$
  where age $$x \ge B$$
5. Because the ratio $\frac{{Y}_{x}}{{Y}_{B}}$ is always $\ge 1$ in our data, we can write this ratio as $1 +\frac{pct}{100}$, where $pct$ is the percentage increase in C/D ratio from age $B$ on the standard C/D ratio scale. Solving Eq. 4 for age $x$, we obtain the following expression:
  5 $$x=\frac{{\text{log}}\left(1+\frac{pct}{100}\right)+R2\cdot B}{R2}$$
  Hence, using Eq. 5, we can estimate the age $x$ at which the C/D ratio is increased by a given $pct$ (on the standard scale) from breakpoint $B$.
